# A Romantic History Connected with a Prominent Dentist

**Published:** 1887-06

**Authors:** 


### Editorial, Etc.

---

A Romantic History Connected with a Prominent Dentist.—The celebrated case of the two dental Parisian-American Evanses which has just ended after twenty years of litigation, during which time Thomas and John Doyle Evans, uncle and nephew, wrestled in the French courts to find what's in a name, is of peculiar interest in Baltimore. It was here that John Evans, the nephew, son of Rodolphe Henri and Elizabeth Evans, first saw and then conquered the affections of his wife, then the handsome Baltimore girl—plain Annie McDonald, now the Marquise Annie Evans d'Oyley. Annie McDonald was the daughter of Alexander McDonald, a printer, who was for some time succeeding 1841 foreman of compositors of the *Sun*. Her mother was the daughter of Thomas Walsh, for years the sexton of the Church of St. Vincent de Paul, on Front street, and also proprietor of a bottling house on South Frederick street. She was baptized in St. Vincent's Church by Rev. Father Myers.

After Mr. McDonald's death Annie and a sister were thrown upon their own resources. Annie, who had been carefully educated in the school attached to St. Vincent's Church, chose to be a governess, and she accepted a position with the family of Mrs. Fitzpatrick, in Montgomery county. It was there that Rev. Edmund Didier, now of St. Vincent's, who was then stationed in Montgomery, became acquainted with Miss McDonald in 1864. Father Didier says the lady, who was a very beautiful brunette, of petite form, was greatly in demand among the society of the place. She was possessed of an exceedingly gentle, generous and religious temperament, coupled with a most lovable and refined disposition, but she was also disposed to be more retiring than the demands upon her presence were willing to concede. In consequence of this she refused many opportunities of appearing in society.

Among her many admirers was Mrs. Elizabeth Evans, mother of John Evans, who kept up an uninterrupted correspondence with her son, who was engaged in dentistry and litigation in Paris. Mrs. Evans persuaded Annie McDonald to take up her residence with her in West Baltimore, and the young lady was adopted as a daughter. Mrs. Evans' glowing description of the graces of her foster daughter were duly sent to her son in France, with urgent appeals that he should come to Baltimore to see his new sister.

John Evans came on in 1870, and met Annie McDonald. It was a true case of love at first sight, and the parties to the romance were wedded shortly afterwards in this city. It was after this marriage that John Evans applied to the Philadelphia Court of Common Pleas to change his name to John d'Oyley a name derived from his middle name, Doyle, when he was made a marquis by Pope Pius IX for his devotion to the church and his celebrity in his chosen profession.

From this circumstance sprang the grounds of the later legal battle between him and his uncle, Thomas Evans, and which has resulted in the French court allowing the younger to practice dentistry under the name of John Evans, and to be known in private life as Marquis d'Oyley. Mrs. John Evans, Marquise d'Oyley, nee Annie McDonald, who left here a purely American girl, with no knowledge of the French language, Father Didier found upon his visits to Paris to be absolutely inured to the French manners of high life, and as fluent in the use of that language as a native. Father Didier says he was overwhelmed with kindness at her beautiful country villa near the French capital, and that he was the recipient of many attentions at the hands of her husband and beautiful children. She presented him with a photograph of herself and three sons in a group, which was countersigned Annie Marquise d'Oyley. Also, a separate portrait of the youngest boy, Alaistre Baron d'Oyley, aged 3½ years. After Mr. Didier's return the Marquise, in honor of her baptism at St. Vincent's, sent a very valuable painting of the Crucifixion, which now adorns the altar place of the sanctuary.

